# Virtual reality as a telerehabilitation strategy for people with autism spectrum disorder during the COVID-19 quarantine scenario: physical activity, motor performance and enjoyment

**DOI:** 10.1080/17483107.2023.2249031

**Published:** 2023-09-11

**Authors:** Amanda Figueiredo Santos de Almeida, Talita Dias da Silva, Íbis Ariana Peña de Moraes, Lilian Del Ciello de Menezes, Eduardo Dati Dias, Luciano Vieira de Araújo, Carlos Bandeira de Mello Monteiro, Helen Dawes, Amanda Orasmo Simcsik, Camila Aparecida de Oliveira Alberissi, Victoria Yanara Hernandes da Silva, Marisa Afonso Andrade Brunherotti, Maria Georgina Marques Tonello

**Affiliations:** a Programa em Promoção de Saúde, Universidade de Franca (Unifran), São Paulo, Brazil; b Grupo de Pesquisa e Aplicações Tecnológicas em Reabilitaçaõ (PATER) da Escola de Artes, Ciências e Humanidades da Universidade de São Paulo (EACH-USP), São, Brazilulo; c Departamento de Medicina (Cardiologia), Escola Paulista de Medicina da Universidade Federal de São Paulo (UNIFESP), São Paulo, Brazil; d Faculdade de Medicina, Universidade Cidade de São Paulo (UNICID). R. Butantã, São Paulo, Brazil; e Faculdade de Medicina, Universidade de São Paulo (USP), Cidade Universitária, São Paulo, Brazil; f NIHR Exeter Biomedical Research Centre, College of Medicine and Health, University of Exeter, Exeter, UK

**Keywords:** Autism spectrum disorder, telerehabilitation, virtual reality, rehabilitation, physical functional performance

## Abstract

**Purpose:**

People with autism spectrum disorder could benefit from physical activity during the pandemic and COVID-19 restrictions, mainly to maintain adequate physical activity. We aimed to evaluate the feasibility, enjoyment, and potential effect of telerehabilitation using a serious game named ‘MoveHero’.

**Materials and methods:**

Registered in Clinical Trials (NCT04402034). We adopted a remotely run Telerehabilitation research design with 44 participants recruited: 22 People with ASD people and 22 non-ASD individuals.

**Results:**

All participants safely participated, 100% adherence to sessions, ∼60% enjoying the task, and significantly improved performance, with better performance for the NA group at most practice moments.

**Conclusions:**

Our findings support both how to implement a gaming intervention and the need to investigate the efficacy of serious games to motivate moderate intensity physical activity in people with ASD.

## IMPLICATIONS FOR REHABILITATION

- A new and thrilling way to promote physical activity is through telerehabilitation to people with Autism Spectrum Disorder.
- A tool that can possibly influence the mood of people with Autism Spectrum Disorder.
- Help to implement home-based rehabilitation to people with Autism Spectrum Disorder.

## Introduction

Autism spectrum disorder (ASD) is characterised by a quantitative deficit in communication and social interaction, along with a series of restricted, repetitive, and stereotyped behaviours and interests [1]. In addition to sensory reactivity, children with ASD have difficulties in visual praxis, somatodyspraxia, and vestibular skills [2]. Characteristics such as changes in muscle tone, fine and gross motor functions, balance, and motor planning are also described with all these factors affecting computer use [3].

Moreover, people with ASD show significative impairments in motor performance and hand motor activities [4] and frequently present a decline in physical activity (PA) level due to a lack of motivation [5], and low interest and enjoyment [6].

Physical activity presents positive effects for people with ASD in sleep quality and cognition [7], and social interaction and communication skills [8], reducing the number of episodes of stereotypical behaviours [9], locomotor skills and muscular strength and endurance [10–13]. Moreover, important factors such as fun, joy, tranquillity, satisfaction, and participation should be considered in all forms of PA for ASD individuals [14].

The impact of the outbreak of COVID-19 as a global pandemic [15] on social distancing, restricting mass gatherings, and encouraging people to stay at home in quarantine [16] has according to the parents of people with ASD been especially challenging and required more commitment than before, with people with ASD being particularly vulnerable to disorganisation from routine interruptions [17]. It is well known that the capability of ASD individuals to be self-sufficient (in functions such as communication, socialisation, daily living, and motor skills) was crucially impaired by the COVID-19 pandemic [18,19]. According to Gregor et al. [20] parents described that the amount of structure within different programs, ratio of staff to participants, location of the activity (indoor/outdoor), use of routines and schedules, acoustics and lighting (sensory inputs) of the space, and type of sport or activity, all influenced PA for ASD Individuals.

Thus, as people with ASD had a high probability of worsening their symptoms [21], interventions through Home-based telerehabilitation (HBTR) could be the most viable possibility to maintain socialisation and communication between the rehabilitation team and patients and could provide physical activity to individuals with disabilities that have parents or caregivers who are not able to travel to a clinic-centre.

HBTR can be defined as a rehabilitation modality in which physicians offer rehabilitation to individuals using telecommunication devices [22]. HBTR can be as effective as conventional rehabilitation in improving activities of daily living, and enhances adherence to rehabilitation training [23,24]. This modality can also make it possible to reduce the therapist and client time, allowing for more regular practice, in addition to facilitating access to on-site rehabilitation centres for those who are unable to travel [25,26]. In ASD, the use of HBTR has been gaining traction globally for the past few decades, bringing positive results in occupational therapy [27], speech-language therapies [28], and approaches for social interactions between the patient, therapist, and their family [29].

Considering different possibilities for organising a HBTR program, a new approach that has been growing in popularity is the use of Virtual Reality (VR) games. VR has been used to elicit light, moderate, and vigorous levels of activity in some patients [30] and has great potential for engaging individuals in physical activity practice [31]. People with ASD are able to use different interfaces and improve performance in a virtual task obtaining significant improvements in physical fitness, executive function, self-perception, and participation [32, 33].

The use of VR systems is increasing in people with ASD [34], with promising improvements shown in both simple skills, such as reaction time [35], and more complex real-life skills, such as pedestrian skills, with higher involvement and motivation and positive transference from the virtual environment to real-life behaviours [36]. According to Karami et al. [37] in a systematic meta-analysis about VR in individuals with ASD, VR technology can be a viable tool for designing interventions aimed at enhancing and improving different skills in people with ASD at any age, with a strong effect observed for daily living skills and a moderate effect for social, communication, and cognitive skills.

Despite these benefits, the use of VR games to improve physical activity in HBTR is still unknown. Thus, the objective of the current study was to test the feasibility of running a trial remotely, during the quarantine period (due to COVID-19), using a VR software platform created with a game that enables the rehabilitation team to manage a physical activity plan, considering the difficulties presented by people with ASD. We hypothesised that all participants would be able to participate safely in the trial, improve performance with the game practice, as well as increased physical activity and enjoyment. However, considering the difficulties that characterise individuals with ASD, we assumed these improvements and benefits would be more evident for the non-ASD group. If this hypothesis is confirmed, the results of this study will be relevant for the use of telerehabilitation as a complementary possibility to provide improvement in performance and physical activities to individuals with ASD.

## Materials and methods

### Study design

Using a cross-sectional design, focused on quantitative data, this study was conducted between March 2020 and July 2020 (quarantine period defined by the state of São Paulo to reduce the transmission of COVID-19) and extended until February 2021. This study follows the guidelines and recommendations of the Strengthening the Reporting of Observational Studies in Epidemiology (STROBE) [38] and was registered in Clinical Trials (NCT04402034). In accordance with the Declaration of Helsinki (1964), later revisions, and the Research Governance Framework for Health and Social Care [39].

### Participant recruitment

**Figure 1.**
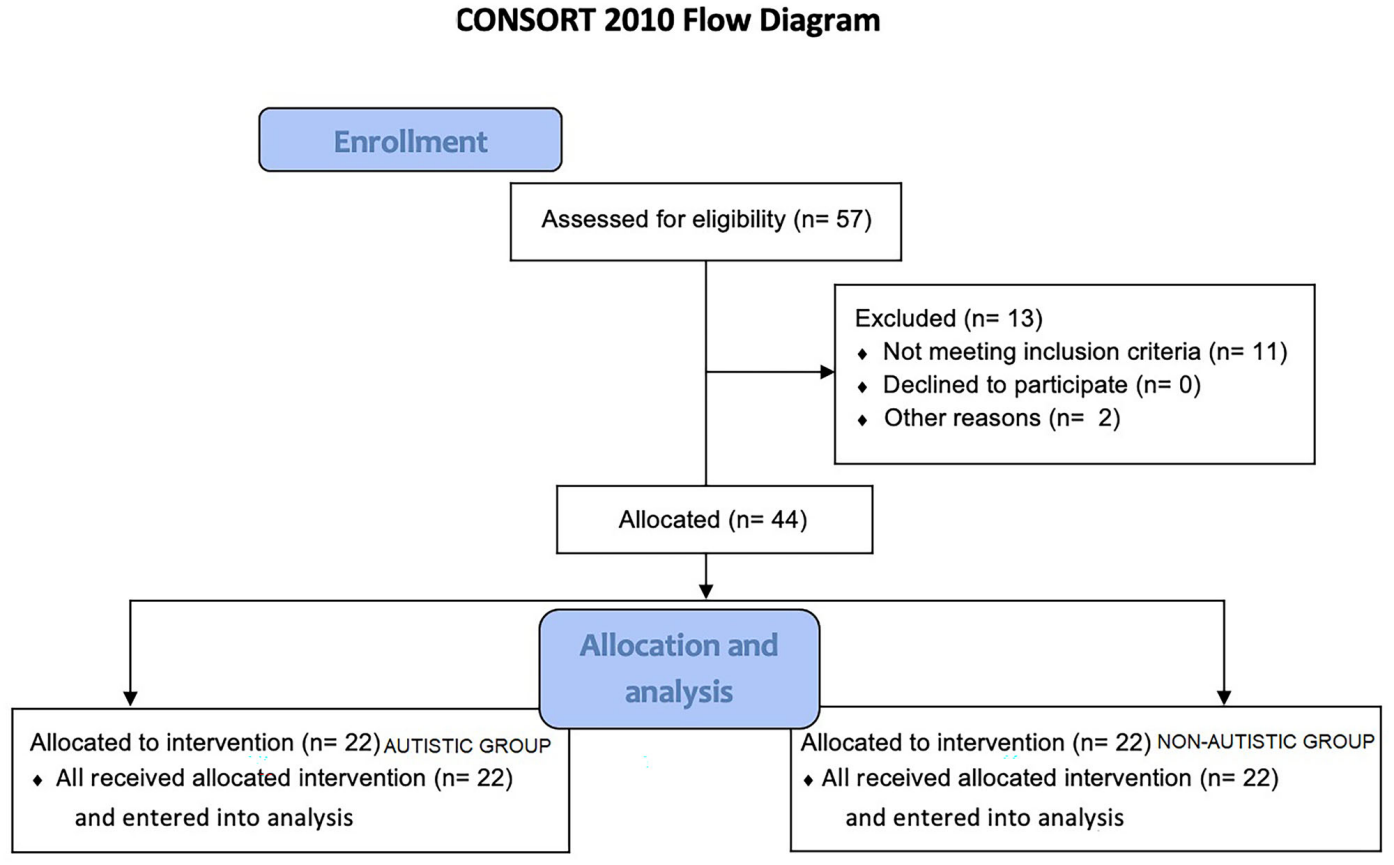
CONSORT 2010 Flow diagram.

The researchers contacted 35 families with children with ASD who usually participated in a rehabilitation program at *Fisioterapia Pediátrica e Cia* (a rehabilitation clinic located in a city in the countryside of São Paulo). After the first contact, during which an explanation of the activity was given, 13 families were unable to participate (10 without a computer or internet connection, 1 without the computer capacity to play the game, and 2 parents without time to help the individuals with ASD. Thus, 22 families agreed to participate in the study. After completion of the intervention by the individuals with ASD, we evaluated 22 non-ASD individuals matched by age and sex with the experimental group. The non-ASD individuals were recruited through disclosure on social media. If they wanted to participate, they got in touch with the researchers and scheduled a day and time to meet online (Figure 1).

#### Inclusion criteria

Young people with ASD were included if they: (1) had a clinical diagnosis of ASD, performed by a neuropediatric doctor or child psychiatrist, (2) were aged from 5 to 13 years, (3) had a light/moderate score on the CARS Scale [40], (4) did not use medication that could interfere with the studied variables, such as cardiac beta-blockers, (5) had access to a computer with a webcam, (6) had an internet connection to use the game, and (7) the individuals assented, and their legal guardians consented to participate in the research. ​​Individuals with low vision or blindness and those who do not have technological devices to perform telerehabilitation were not included in the study.

#### Exclusion criteria

Participants were excluded if (1) they did not understand the tasks (comprehension of the task was assessed through 2 min of practicing the task, each match lasted 5 min, if the patient could complete the match without missing more than 10 spheres continuously, they were allowed to move on to the next match; (2) they had difficulties in maintaining attention and self-regulation that prevented the completion of virtual tasks; (3) they do not have appropriate devices to perform the virtual task (computer) or to contact the researcher (cell phone or other computer); (4) the system did not work during practice due to some technological failure (such as internet connection failure).

### Material and apparatus

#### Instrument

**Figure 2.**
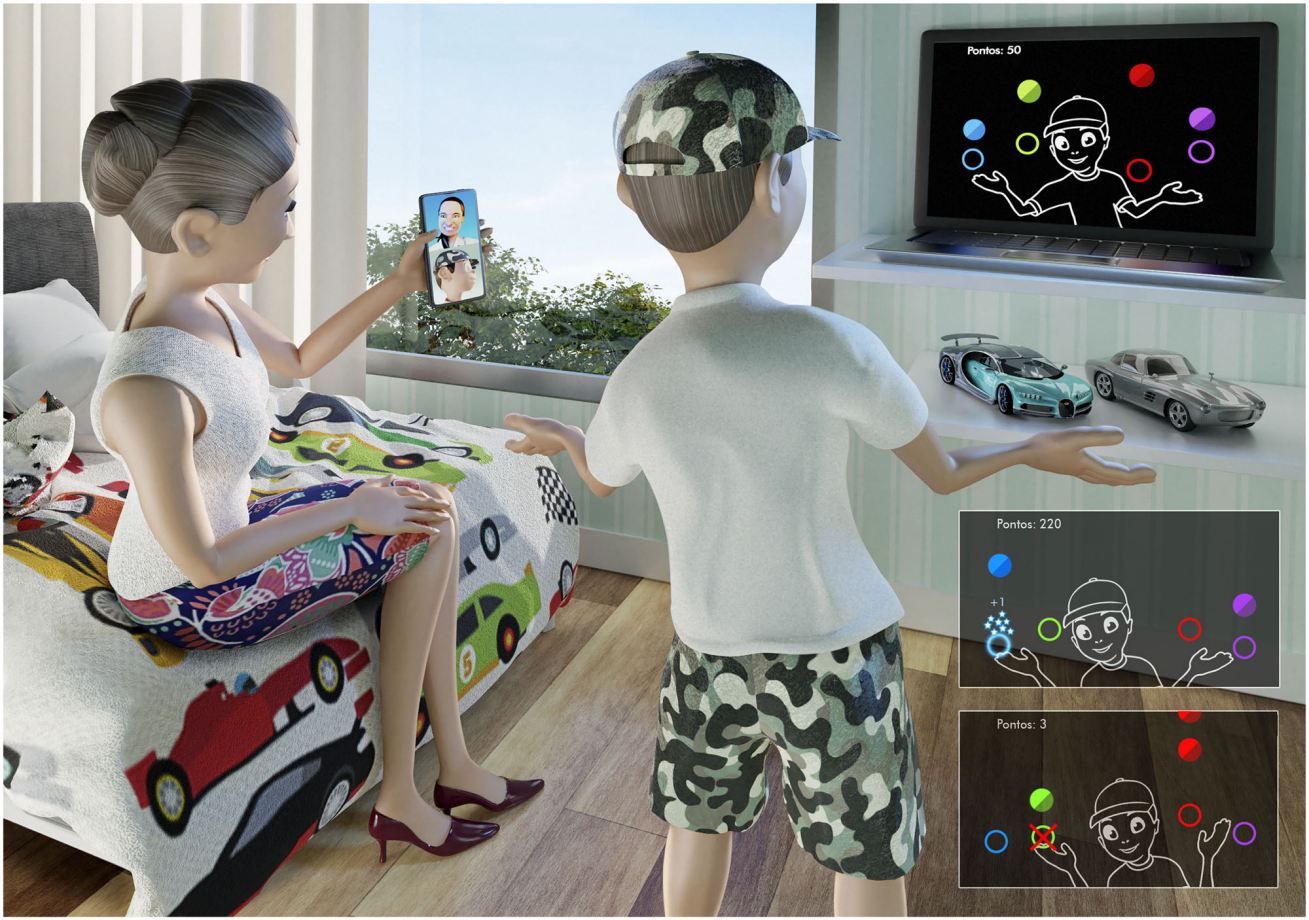
Participant Positioning and game design. The task was to intercept different falling spheres using upper limb wave movements at the exact moment the spheres reached their specific target. Bottom right image, miss feedback - above this one, hit feedback. The game is available for free use in Portuguese https://movehero.com.br/ and English https://movehero.com.br/en/.

In the current study, we used a platform called MoveHero (Figure 2), available for free use in Portuguese https://movehero.com.br/ and English https://movehero.com.br/en/. As presented by Martins et al. [41], MoveHero is a coincident timing task and consists of several spheres falling down the computer screen, with a musical rhythm to increase engagement. The participant is positioned in front of a computer and when the game starts the webcam captures the participant’s movements and a representation of the player appears on the computer screen as an avatar (for more information about the game see Dias et al. [42, 43], Silveira et al. [44], Moraes et al. [45] and Ribeiro [46]).

#### Assessments

To characterise the participants, we used:

1. the Childhood Autism Rating Scale – CARS [40] (parent-proxy report), to assess the severity of the autism, classified as follows: a total score of 15–29.5 was considered ‘non-autistic,’ a score of 30–36.5 was considered ‘mild to moderate’ autism, and a score of 37–60 was considered ‘moderate to severe’ autism [47]. This scale presented internal consistency and sensitivity, and should be used along with other confirmatory tools [48].
2. Sociodemographic information (parent-proxy report) was collected through a questionnaire, used to assess information such as sex, if they had feelings of loneliness, trouble sleeping, if the participant practiced physical activity, and the use of games in their routine.

To analyse the Outcome, we used:

1. the Brunel Mood Scale – BRUMS [49,50] (participant report), this scale contains 24 simple mood indicators, such as feelings of anger, disposition, nervousness, and dissatisfaction that are noticeable by the individual being assessed. The possible score is 5 points according to how they feel about these sensations (0 = nothing to 4 = extreme). The scale takes about 1 to 2 min to complete. The 24 indicators of the scale comprise six subscales: anger, confusion, depression, fatigue, tension, and vigour [51,52].
2. Enjoyment Scale (participant report), using smiley faces (0 ‘not fun at all,’ 1 ‘boring,’ 2 ‘a little bit fun,’ 3 ‘fun,’ and 4 ‘great fun’) was applied after the telerehabilitation, to verify the participant’s level of enjoyment while performing the activity. This scale was developed to evaluate how people feel when interacting with the proposed non-immersive VR games and was previously used in other studies [53].
3. The Rating of Perceived Exertion – CR10 (RPE) [54] (participant report), is a tool for monitoring the intensity of physical effort. The participant is required to score their feeling of tiredness from 0 to 10. This scale is used both in areas of high-performance sports and physical rehabilitation to monitor the changes caused by physical exercise in the cardiorespiratory, metabolic, and neuromuscular systems [55,56].

#### Procedure and design

The guardians of the participants were contacted by phone and asked to help the individuals with ASD to perform telerehabilitation sessions at home, led by the researcher over the phone, with a video call. During the video call, all the instructions on how to conduct the game and tasks were given to the parents and guardians. The guardians expressing an interest in the study received a link with the assent and consent forms to fill out and to complete a Google Forms survey for all measures.

### Protocol

**Figure 3.**
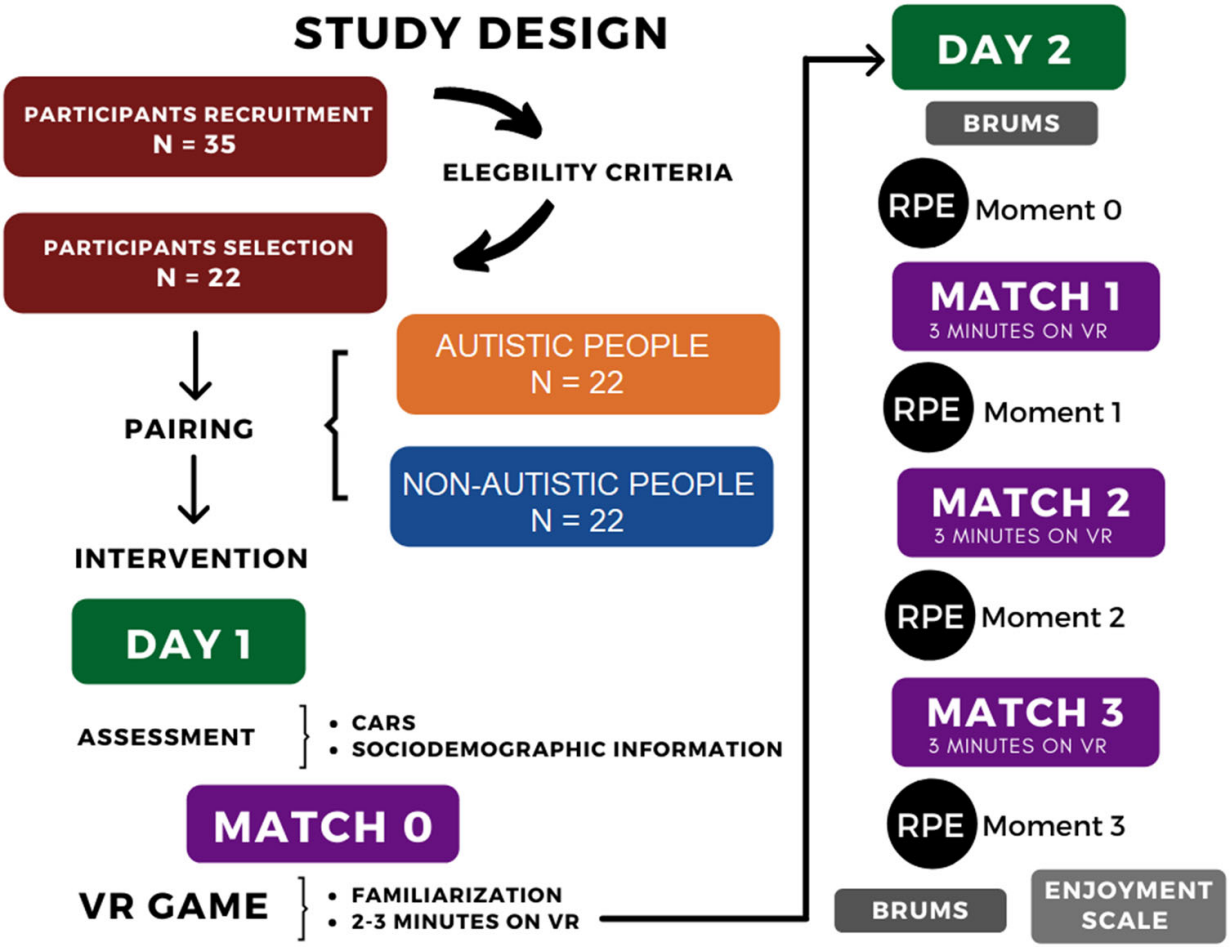
Study design.

The protocol was divided into 4 matches where M0 was considered the first contact with the task (first day), when the participants gained understanding of the game (familiarisation match) by playing for 3 min. After the familiarisation and on a second day all participants answered the BRUMS and played matches M1, M2, and M3 (all three matches consisted of 3 min playing the game) with intervals of approximately 20 s between matches to receive the score and answer the RPE scale. After the 3 matches the BRUMS was answered again and the enjoyment scale was applied (Figure 3).

To determine the sample size, we used statistical software (G∗Power 3.1.5) on the main outcome measure (i.e., the motor score). This calculation was based on data from five patients (pilot study). The power was 0.80; the α was 0.05, and the effect size was 0.65 (Cohen d). The sample estimation indicated that 40 participants would be necessary (i.e., 20 per group), and with an adjustment to allow for a withdrawal rate (20%), we aimed to recruit 24 participants per group, considering the intrinsic needs of the research, such as internet connection, computer capacity to play the game, and parents with time to help the participants with ASD, we contacted 35 families of individuals with ASD, to get 22 participants with ASD.

### Community involvement statement

The researchers recruited an advisory board that comprised people with ASD, parents of children with ASD, and two clinical practitioners, all related to the clinic Fisioterapia Pediátrica e Cia, a rehabilitation clinic located in a city in the countryside of São Paulo.

### Data Analysis

The MoveHero game is considered a timing coincident task that provides a dependent variable with timing error (i.e., timing error was defined as the difference between the time when the sphere started dropping and the time the individual managed to hit the target with the avatar’s hand). Thus, using MoveHero we can verify two important movement errors: 1) absolute error (AE), which demonstrates the accuracy of the movement, calculated by taking the absolute value of each raw score and disregarding whether the response was early or late, and 2) variable error (VE), which identifies the precision of the movement, calculated as the timing error resulting from within-subject variability. The calculation considered the square root of the sum of the square of differences between each score and the individual constant error mean divided by the number of trials (for details about these errors, see de Mello Monteiro et al. [57] and Moraes et al. [33]).

The absolute error (AE), variable error (VE), and percentage of hits and misses were submitted to a Multiple Analysis of Variance with factors: 2 (Groups: autistic and non-autistic) by 4 (Matches: M0, M1, M2, and M3), by 4 (Target Positions: P1, P2, P3, and P4) with repeated measures for Matches and Target Positions, as well as Physical Activity as a covariate (MANCOVA). Rating of Perceived Exertion (RPE) was submitted to Analysis of Variance (ANOVA); with factors 2 (Groups: autistic and non-autistic) by 4 (Moments: Mo0, Mo1, Mo2, and Mo3). The Enjoyment Scale was submitted to a chi-square test using the variables Groups (2: autistic and non-autistic) and how fun the game was (2: Less fun, More fun). Brunel Mood Scale data of 6 dimensions (Anger, Confusion, Depression, Fatigue, Tension, Vigour) were submitted to a paired samples t-test. The Least Significant Difference (LSD) was used as a post-hoc test. The partial eta-squared (ηp^2^) was reported to measure the effect size and interpreted as small (effect size> 0.01), medium (effect size> 0.06), or large (effect size> 0.14) [58]. We also reported the Observed power (op). Values ​​of *p* < 0.05 were considered significant. The statistical package used was the Statistical Package for Social Sciences (SPSS; IBM, Chicago, Illinois, USA), version 26.0.

## Results

A total of 44 individuals participated in this study (22 individuals with ASD; 22 non-ASD). The mean age of the participants with ASD was 8.05 ± 2.3 (5 to 13 years old) and non-ASD was 8.95 ± 2.4, with three girls (13.5%) and 19 boys (86.5%) in each group. Regarding the CARS, the mean score of the ASD group was 32.8 ± 2.3. Data about participant’s characteristics are presented in Table 1.

**Table 1.** Participant’s characteristics.

Variables		Group		*p-value*
		ASD	NON-ASD	
		*Mean ± Standard Deviation*	*Mean ± Standard Deviation*	
		*n (%)*	*n (%)*	
Sex	Female	3 (13.6)	5 (22,7)	0.698
	Male	19 (86.4)	17 (77.3)	
I feel a lot of loneliness during quarantine. How much you agree with this sentence.	I disagree	2 (66.7)	1 (33.3)	
	I Partially Agree	12 (60.0)	8 (40.0)	0.00
	I completely agree	8 (38.1)	13 (61.9)	
Regarding sleep, do you have difficulty sleeping?	Neither	9 (39.1)	14 (60.9)	
	Light	8 (50.0)	8 (50.0)	0.00
	Moderate	5 (100)	0 (0.0)	
Do you practice physical activity?	Once a week	13 (61.9)	8 (38.1)	
	between 2 and 4 times a week	9 (45.0)	11 (55.0)	0.00
	5 times a week or more	0 (0.0)	3 (100)	
If YES, did you have to stop physical activities during the quarantine?	No, I adapted the activities at home	5 (50.0)	5 (50.0)	1.00
	Yes	17 (50.0)	17 (50.0)	
Would you use this game in a physical therapy session?	No	4 (100)	0 (0,0)	0.108
	Yes	18 (45.0)	22 (55.0)	
Do you play any games on your cell phone?	No	2 (50.0)	2 (50.0)	1.000
	Yes	20 (50.0)	20 (50.0)	

As we found a significant difference between groups regarding physical activity, we first ran the MANCOVA, using physical activity as a covariate, but we did not find any effect for this factor. Thus, we proceeded to the results taking physical activity as a covariate. For Absolute and Variable errors, the MANOVA found a significant effect for Matches [Wilks’ lambda = 0.295, F_6, 13_ = 5.18, *p* = 0.006, η_p_^2^ = 0.71, op = 0.93] with no further effects or interactions. The separate ANOVAs are described in the sections below:

### Absolute error (AE)

**Figure 4.**
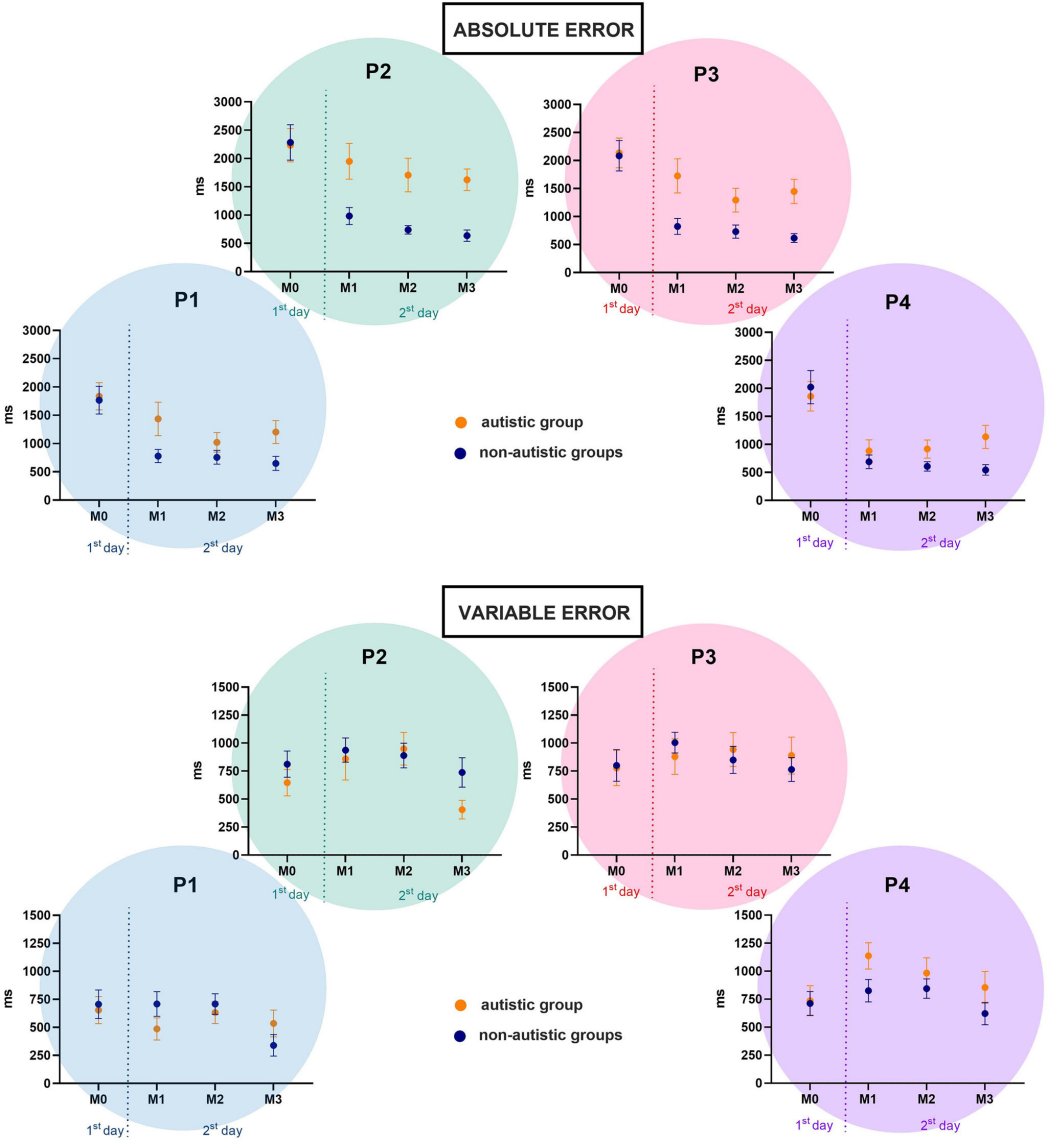
Absolute error and variable error mean and standard error for both ASD and non-ASD groups.

There were significant main effects for Matches [F_3, 54_ = 14.3, *p* < 0.001, η_p_^2^ = 0.44, op = 0.99] and Target Positions [F_3, 54_ = 4.15, *p* = 0.024, η_p_^2^ = 0.19, op = 0.69]. The post-hoc tests showed that there was improvement from M0 (*m* = 1868 ms) to M1 (*m* = 1079 ms; *p* = 0.003), M2 (*m* = 968 ms; *p* < 0.001), and M3 (*m* = 824 ms; *p* < 0.001), as well as from M1 to M3 (*p* = 0.050). Regarding the Target Position, although there was no significant interaction between Groups and Target Position, the *post hoc* test showed that participants from the ASD group presented better performance in the lateral targets (P1 = 1235 ms and P4 = 1087 ms; against P2 = 1733 ms and P3 = 1447 ms), while the non-ASD group presented better performance in the right targets (P3 = 988 ms and P4 = 770 ms; against P1 = 1142 ms and P2 = 1077 ms). The mean and standard errors are depicted in Figure 4.

### Variable error (VE)

There were significant main effects for Matches [F_3, 54_ = 4.37, *p* = 0.013, η_p_^2^ = 0.20, op = 0.85] and a significant interaction between Groups, Matches, and Target Positions [F_9, 162_ = 2.21, *p* = 0.050, η_p_^2^ = 0.11, op = 0.72]. The post-hoc tests showed that there was improvement from M0 (*m* = 1868 ms) to M1 (*m* = 1079 ms; *p* = 0.003), M2 (*m* = 968 ms; *p* < 0.001), and M3 (*m* = 824 ms; *p* < 0.001), as well as from M1 to M3 (*p* = 0.050).

Regarding the interaction between Group, Matches, and Target Position, the *post hoc* test showed that for the ASD group the improvement from M0 to M1 only occurred for Positions 2 (*p* = 0.009) and 3 (*p* = 0.009), with no significant improvements from M0 to M2, and from M0 to M3 the improvement was significant for Target Positions 2 (*p* = 0.009) and 4 (*p* = 0.005). For the non-ASD group, there were no significant improvements between any matches (Figure 4).

### Percentage of hits and misses

Regarding Hits, there were significant main effects for Matches [F_3, 111_ = 36.8, *p* < 0.001, η_p_^2^ = 0.50, op = 1.0] and marginally significant effects for Groups [F_1, 37_ = 3.81, *p* = 0.058, η_p_^2^ = 0.09, op = 0.09], as well as a significant interaction between Groups and Matches [F_3, 111_ = 4.69, *p* = 0.018, η_p_^2^ = 0.11, op = 0.72]. The *post hoc* test showed that both groups presented significant improvements in the percentage of hits from M0 to all other Matches (M1: *p* < 0.001; M2: *p* < 0.001; M3: *p* < 0.001). The *post hoc* for the interaction between Groups and Matches showed that the differences between groups were present in M1 (*p* = 0.040), M2 (*p* = 0.066) and M3 (*p* = 0.006), but not in M0.

**Figure 5.**
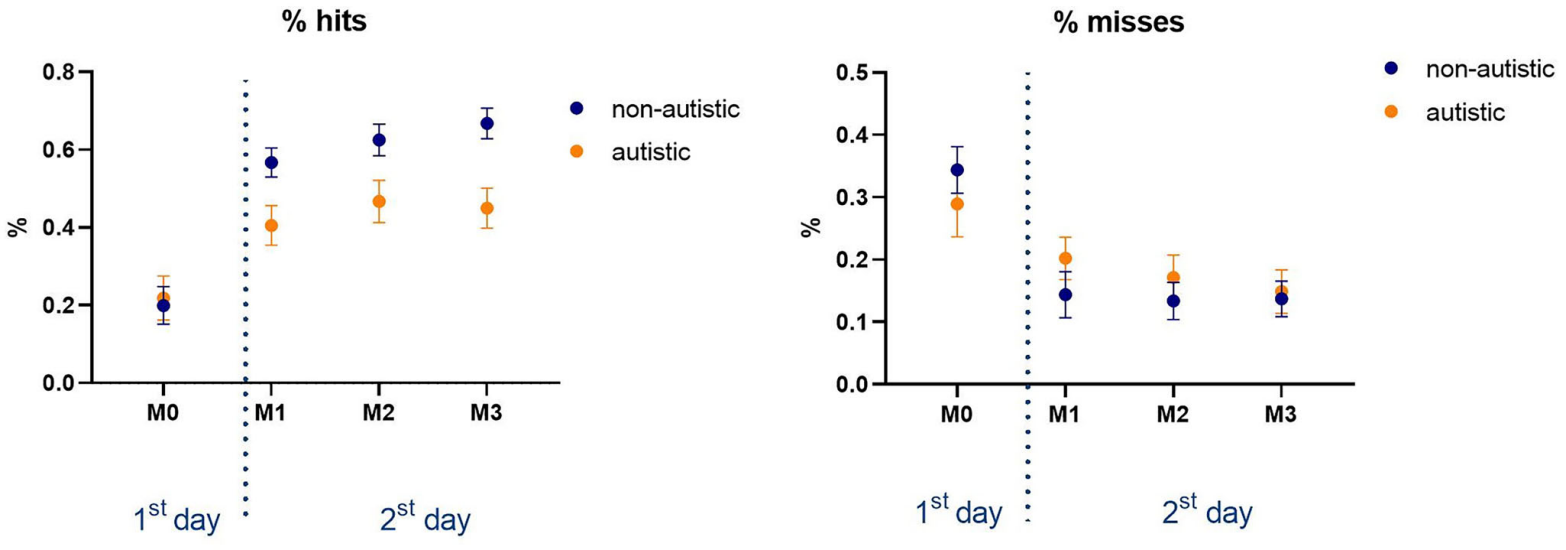
Mean and standard error of percentage of hits, misses and the continuous score, for both ASD and non-ASD groups.

Considering Misses, only a main effect for Matches [F_3, 54_ = 1.97, *p* < 0.001, η_p_^2^ = 0.39, op = 1.0] was found, and similarly to Hits, the *post hoc* test showed that both groups significantly decreased the percentage of misses from M0 to all other Matches (M1: *p* < 0.001; M2: *p* < 0.001; M3: *p* < 0.001). The means and standard errors are presented in Figure 5.

### Rating of Perceived Exertion (RPE) - exercise intensity and enjoyment Scale

**Figure 6.**
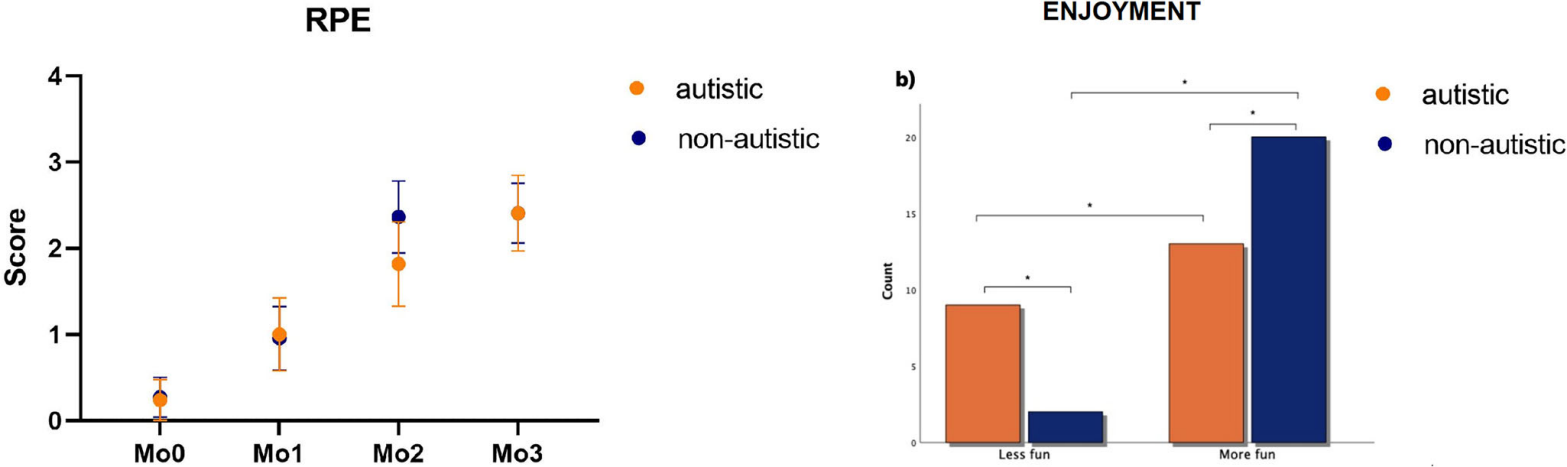
Mean and standard error of RPE score (a) and representation of the answers of the individuals regarding their enjoyment with the game (b) for both ASD and non-ASD groups.

There was only a main effect for Moments [F_3, 123_ = 15.0, *p* < 0.001, η_p_^2^ = 0.27, op= 1.0]. The *post hoc* test showed that both groups significantly increased the RPE from Mo0 to all other Moments (Mo1: *p* = 0.025; Mo2: *p* < 0.001; Mo3: *p* < 0.001), with no significant differences between groups (Figure 6a). The level rose from very light to moderate intensities (1-3).

Regarding their enjoyment (Figure 6b), a significantly higher number of participants from both ASD and non-ASD groups found the intervention more fun than less fun (Pearson chi-square: *p =* 0.015)

### Brunel Mood Scale (BRUMS)

The data on mood before and after the intervention are described in Table 2.

**Table 2.** Brunel Mood Scale (BRUMS) for both groups, before and after the intervention.

	ASD			NON-ASD		
	*before*	*After*	*p-value*	*Before*	*after*	*p-value*
Tension	**0.69**	**0.50**	***0.014***	**0.48**	**0.20**	***<0.001***
Depression	0.28	0.28	*1.000*	0.06	0.04	*0.329*
Anger	**0.26**	**0.76**	***0.020***	0.00	0.09	*0.329*
Vigor	**3.36**	**3.98**	***0.049***	3.40	3.33	*0.565*
Fatigue	**0.03**	**0.29**	***0.001***	0.01	0.05	*0.266*
Mental Confusion	0.45	0.41	*0.683*	0.08	0.07	*0.576*
Total	0.88	0.91	*0.536*	**0.71**	**0.64**	***<0.001***

Legend: Table 2 represents the comparison between groups and moment of assessment (pre and post intervention) of mood using the Brunel Mood Scale. A value of *p* < 0.05 is considered significant.

## Discussion

The present study investigated the possibility of running a trial in telerehabilitation using a VR platform and found it possible for two-thirds of invited families to participate and for the intervention to be safe and feasible for participating children with ASD. We found that all participants presented improved performance on repeated trials, with better performance for the non-ASD group at most practice moments. Importantly, most participants (∼60%) enjoyed the games, and repeated exposures to the games demonstrated very high levels of enjoyment, with all participants completing all sessions, and exercising at exercise intensities reflective of moderate intensities at the later sessions.

We were able to observe factors that made the game easier or harder for people with ASD, such as the position of the targets. Taken together, our findings are exciting and support both how to implement a gaming intervention and the need for a remote trial to investigate the efficacy of serious games to motivate moderate intensity physical activity over the long term in people with ASD. Some important findings are presented below.

### Performance improvement

The individuals with ASD presented similar movement accuracy (absolute error) and precision (variable error) to non-ASD individuals at the familiarisation moment in all positions (M0 – first day). After the familiarisation, both groups showed improvement in performance during the game considering absolute error, however, the non-ASD group performed better. This result was expected as studies using VR in individuals with ASD identified improvement in motor skills for them, as well as better performance for non-ASD groups [33,35], likely due to altered sensorimotor control and a greater reliance on proprioceptive and tactile information in individuals with ASD [59,60].

Another novel observation in performance was that the ASD group had a higher performance (i.e., they hit a larger number of spheres) on the external targets of the game with the same performance as the non-ASD group in absolute error. One factor that may have contributed to this result is the common excessive activity in individuals with ASD and the difficulty in reaching or crossing the middle line, which could facilitate distal practice (i.e., distal movements have benefits with more range of movement) [61,62].

### Physical activity (PA)

We believe that the most important result from our study was the increase in the rating of perceived exertion (RPE scale) continuously during the practice in the VR game for both groups where intensity levels were moderate for most in later sessions. Suggesting gaming is an effective approach to motivate people with ASD to exercise at moderate levels and possibly achieve health and well-being benefits.

Before the pandemic, the use of online technologies was not a usual practice among children [7], while now, children and their parents or caregivers are more receptive to this kind of practice, especially those who have limited mobility or live far from the places they used to go to receive treatment. Sorensen & Zarett [63] in a review about the benefits of physical activity found strong evidence that physical activity can be effective in clinical motor signs presented in individuals with ASD. We agree with the statement from Cynthia et al. [64] that enhancing motor coordination and motor skills may not only benefit PA participation, but also translate to other aspects of daily living, such as social skills and manual dexterity. Thus, our results can be considered encouraging and may positively influence rehabilitation professionals prescribing telerehabilitation to people with Autism Spectrum Disorder, providing an effective alternative to promote PA, since autistic individuals with ASD prefer videogames for recreational activity, and tend to play with high frequency.

### Enjoyment

In the pandemic scenario, parents of people with ASD reported that the anxiety, fear, stress, low mood and difficulty in emotion management were symptoms that their children were facing during the isolation time [65, 66]. With the possibility of changing their humour by giving a protocol that could promote motivation, another important result of our study, which may be responsible for the improvement in performance and increase in PA was the impact of enjoyment using the VR game during HBTR. The majority of ASD group (59.5%) and non-ASD group participants (91%) characterised the task as ‘fun’ or ‘very fun’.

Tse et al. [7] demonstrates that children with ASD struggle with self-regulation of anger that can be considered a characteristic of people with ASD when practicing PA. Individuals with ASD presented reduced tension and increased vigour after practice with the MoveHero game, which could suggest that this platform provides the expected positive changes in mood from physical activity, although we also found increases in anger and fatigue after the practice. Therefore, considering the decline in PA levels in ASD related to factors such as lack of motivation, low interest, and low enjoyment [5,6,45,67,68], our results can be considered evidence of an interesting tool that facilitates the use of technology to promote PA, making it attractive and enjoyable for individuals with ASD.

### Limitations

Although we found interesting results, we can point out some limitations of the present study: (1) this was a one-day protocol and a protocol with a training program should provide important information and continuous benefits to individuals with ASD; (2) we did not analyse specific motor and cognitive abilities because they are difficult to assess in a telerehabilitation protocol; (3) we did not analyse the type of music used (which could influence the performance of ASD group); and (4) we should have measured the influence of the therapist and family members during the practice, as these data could be important to define future interventions;

## References

[1] American Psychiatric Association. Diagnostic and statistical manual of mental disorders., 5th ed.; Philadelphia, PA, USA:APA; 2013.

[2] Roley SS, Mailloux Z, Parham LD, et al. Sensory integration and praxis patterns in children with autism. Am J Occup Ther. 2015;69(1):6901220010–6901220010p8. doi:10.5014/ajot.2015.012476. 25553746

[3] Miller M, Chukoskie L, Zinni M, et al. Dyspraxia, motor function and visual-motor integration in autism. Behav Brain Res. 2014;269:95–102. doi:10.1016/j.bbr.2014.04.011. 24742861 PMC4072207

[4] De Luca R, Leonardi S, Portaro S, et al. Innovative use of virtual reality in autism spectrum disorder: a case-study. Appl Neuropsychol Child. 2021;10(1):90–100. doi:10.1080/21622965.2019.1610964. 31092007

[5] Stanish H, Curtin C, Must A, et al. Enjoyment, barriers, and beliefs about physical activity in adolescents with and without autism spectrum disorder. Adapt Phys Activ Q. 2015;32(4):302–317. doi:10.1123/APAQ.2015-0038. 26485735 PMC4766586

[6] Obrusnikova I, Cavalier AR. Perceived barriers and facilitators of participation in after-School physical activity by children with autism spectrum disorders. J Dev Phys Disabil. 2011;23(3):195–211. doi:10.1007/s10882-010-9215-z.

[7] Tse C, Lee HP, Chan K, et al. Examining the impact of physical activity on sleep quality and executive functions in children with autism spectrum disorder: a randomised controlled trial. Autism. 2019;23(7):1699–1710. doi:10.1177/1362361318823910. 30663324

[8] Zhao M, Chen S. The effects of structured physical activity program on social interaction and communication for children with autism. Biomed Res Int. 2018;2018:1825046. doi:10.1155/2018/1825046. 29568743 PMC5820623

[9] Ferreira JP, Ghiarone T, Júnior C, et al. Effects of physical exercise on the stereotyped behavior of children with autism spectrum disorders. Medicina. 2019;55(10):685. doi:10.3390/medicina55100685. 31615098 PMC6843401

[10] Pan CY, Chu CH, Tsai CL, et al. The impacts of physical activity intervention on physical and cognitive outcomes in children with autism spectrum disorder. Autism. 2017;21(2):190–202. doi:10.1177/1362361316633562. 27056845

[11] Rafie F, Ghasemi A, Zamani Jam A, et al. Effect of exercise intervention on the perceptual-motor skills in adolescents with autism. J Sports Med Phys Fitness. 2017;57(1–2):53–59. doi:10.23736/S0022-4707.16.05919-3. 27028719

[12] Healy S, Nacario A, Braithwaite RE, et al. The effect of physical activity interventions on youth with autism spectrum disorder: a meta-analysis. Autism Res. 2018;11(6):818–833. doi:10.1002/aur.1955. 29693781

[13] Ruggeri A, Dancel A, Johnson R, et al. The effect of motor and physical activity intervention on motor outcomes of children with autism spectrum disorder: a systematic review. Autism. 2020;24(3):544–568. doi:10.1177/1362361319885215. 31782658

[14] Jachyra P, Renwick R, Gladstone B, et al. Physical activity participation among adolescents with autism spectrum disorder. Autism. 2021;25(3):613–626. doi:10.1177/1362361320949344. 32921151

[15] Adil MT, Rahman R, Whitelaw D, et al. SARS-CoV-2 and the pandemic of COVID-19. Postgrad Med J. 2021;97(1144):110–116. doi:10.1136/postgradmedj-2020-138386. 32788312 PMC10016996

[16] Wiersinga WJ, Rhodes A, Cheng AC, et al. Transmission, diagnosis, and treatment of coronavirus disease 2019 (COVID-19): a review. JAMA. 2020;324(8):782–793. 32648899 10.1001/jama.2020.12839

[17] Colizzi M, Sironi E, Antonini F, et al. Psychosocial and behavioral impact of COVID-19 in autism spectrum disorder: an online parent survey. Brain Sci. 2020;10(6):341. doi:10.3390/brainsci10060341. 32503172 PMC7349059

[18] Valenti M, Pino MC, Le Donne I, et al. Adaptive response of Italian young adults with autism to the COVID-19 pandemic: a longitudinal study. Res Dev Disabil. 2022;131:104333. doi:10.1016/j.ridd.2022.104333. 36162352 PMC9464572

[19] Siracusano M, Segatori E, Riccioni A, et al. The impact of COVID-19 on the adaptive functioning, behavioral problems, and repetitive behaviors of Italian children with autism spectrum disorder: an observational study. Children. 2021;8(2):96. doi:10.3390/children8020096. 33540683 PMC7913091

[20] Gregor S, Bruni N, Grkinic P, et al. Parents’ perspectives of physical activity participation among Canadian adolescents with autism spectrum disorder. Res Autism Spectr Disord. 2018;48:53–62. doi:10.1016/j.rasd.2018.01.007.

[21] Panda PK, Gupta J, Chowdhury SR, et al. Psychological and behavioral impact of lockdown and quarantine measures for COVID-19 pandemic on children, adolescents and caregivers: a systematic review and meta-analysis. J Trop Pediatr. 2021;67(1):fmaa122. doi:10.1093/tropej/fmaa122. 33367907 PMC7798512

[22] Chen J, Sun D, Zhang S, et al. Effects of home-based telerehabilitation in patients with stroke: a randomized controlled trial. Neurology. 2020;95(17):e2318–e2330. doi:10.1212/WNL.0000000000010821. 32999058

[23] Ott KK, Schein RM, Straatmann J, et al. Development of a home-based telerehabilitation service delivery protocol for wheelchair seating and mobility within the veterans health administration. Mil Med. 2022;187(5–6):e718–e725. doi:10.1093/milmed/usab091. 33647955

[24] Saito T, Izawa KP. Effectiveness and feasibility of home-based telerehabilitation for community-dwelling elderly people in Southeast Asian countries and regions: a systematic review. Aging Clin Exp Res. 2021;33(10):2657–2669. doi:10.1007/s40520-021-01820-3. 33765258 PMC7993072

[25] Szturm T, Imran Z, Pooyania S, et al. Evaluation of a game based tele rehabilitation platform for in‐home therapy of hand‐arm function post stroke: feasibility study. Pm R. 2021;13(1):45–54. doi:10.1002/pmrj.12354. 32107868

[26] Lloréns R, Noé E, Colomer C, et al. Effectiveness, usability, and cost-benefit of a virtual reality–based telerehabilitation program for balance recovery after stroke: a randomized controlled trial. Arch Phys Med Rehabil. 2015;96(3):418–425.e2. doi:10.1016/j.apmr.2014.10.019. 25448245

[27] Gibbs V, Toth-Cohen S. Family-centered occupational therapy and telerehabilitation for children with autism spectrum disorders. Occup Ther Health Care. 2011;25(4):298–314. doi:10.3109/07380577.2011.606460. 23899082

[28] Karrim SB, Flack PS, Naidoo U, et al. The experiences of speech-language therapists providing telerehabilitation services to children with autism spectrum disorder. S Afr J Commun Disord. 2022;69(2):e1–e12. doi:10.4102/sajcd.v69i2.917. PMC9453137 36073081

[29] Vallefuoco E, Purpura G, Gison G, et al. A multidisciplinary telerehabilitation approach for supporting social interaction in autism spectrum disorder families: an Italian digital platform in response to COVID-19. Brain Sci. 2021;11(11):1404. doi:10.3390/brainsci11111404. 34827403 PMC8615374

[30] Gomes TT, Schujmann DS, Fu C. Rehabilitation through virtual reality: physical activity of patients admitted to the intensive care unit. Braz J Intensive Care. 2019;31(4):456–463. doi:10.5935/0103-507X.20190078 PMC7008986 31967219

[31] Farič N, Smith L, Hon A, et al. Virtual reality exergame to engage adolescents in physical activity: mixed methods study describing the formative intervention development process. J Med Internet Res. 2021;23(2):e18161. doi:10.2196/18161. 33538697 PMC7892288

[32] Fang Q, Aiken CA, Fang C, et al. Effects of exergaming on physical and cognitive functions in individuals with autism spectrum disorder: a systematic review. Games Health J. 2019;8(2):74–84. doi:10.1089/g4h.2018.0032. 30332294

[33] Moraes ÍAP, Monteiro CBM, Silva TD, et al. Motor learning and transfer between real and virtual environments in young people with autism spectrum disorder: a prospective randomised cross over controlled trial. Autism Res. 2020;13(2):307–319. doi:10.1002/aur.2208. 31566888

[34] Mesa-Gresa P, Gil-Gómez H, Lozano-Quilis JA, et al. Effectiveness of virtual reality for children and adolescents with autism spectrum disorder: an evidence-based systematic review. Sensors. 2018;18(8):2486. doi:10.3390/s18082486. 30071588 PMC6111797

[35] Herrero D, Crocetta TB, Massetti T, et al. Total reaction time performance of individuals with autism after a virtual reality task. Int J Neurorehabilitation Eng. 2015;02(05):1–5. doi:10.4172/2376-0281.1000189.

[36] Saiano M, Garbarino E, Lumachi S, et al. Effect of interface type in the VR-based acquisition of pedestrian skills in persons with ASD Annual International Conference of the IEEE Engineering in Medicine and Biology Society. IEEE Engineering in Medicine and Biology Society. Annual International Conference. 2015;p. 5728–5731. 10.1109/EMBC.2015.7319693 26737593

[37] Karami B, Koushki R, Arabgol F, et al. Effectiveness of virtual/augmented reality-based therapeutic interventions on individuals with autism spectrum disorder: a comprehensive meta-analysis. Front Psychiatry. 2021;12:665326. doi:10.3389/fpsyt.2021.665326. 34248702 PMC8260941

[38] von Elm E, Altman DG, Egger M, et al. The strengthening the reporting of observational studies in epidemiology (STROBE) statement: guidelines for reporting observational studies. PLOS Med. 2007;4(10):e296. doi:10.1371/journal.pmed.0040296. 17941714 PMC2020495

[39] Taylor M. Research governance framework for health and social care. Health Soc Care Community. 2002;10(1):6–6. doi:10.1046/j.0966-0410.2001.00336.x-i1. 11892625

[40] Schopler E, Reichler RJ, DeVellis RF, et al. Toward objective classification of childhood autism: childhood autism rating scale (CARS). J Autism Dev Disord. 1980;10(1):91–103. doi:10.1007/BF02408436. 6927682

[41] Martins F, Massetti T, Crocetta TB, et al. Analysis of motor performance in individuals with cerebral palsy using a non-immersive virtual reality task - a pilot study. Neuropsychiatr Dis Treat. 2019;15:417–428. doi:10.2147/NDT.S184510. 30787616 PMC6366350

[42] da Silva TD, da Silva PL, Valenzuela EdJ, et al. Serious game platform as a possibility for home-based telerehabilitation for individuals with cerebral palsy during COVID-19 Quarantine - A cross-sectional pilot study. Front Psychol. 2021;12:622678. doi:10.3389/fpsyg.2021.622678. 33633648 PMC7901904

[43] Silva TD, Fontes AMGG, de Oliveira-Furlan BS, et al. Effect of combined therapy of virtual reality and transcranial direct current stimulation in children and adolescents with cerebral palsy: a study protocol for a triple-blinded randomized controlled crossover trial. Front Neurol. 2020;11:953. doi:10.3389/fneur.2020.00953. 32982950 PMC7492207

[44] Silveira AC, Moraes ÍA, Vidigal GP, et al. Cardiac autonomic modulation in subjects with amyotrophic lateral sclerosis (ALS) during an upper limb virtual reality task: a prospective control trial. Biomed Res Int. 2022;2022:4439681–4439611. doi:10.1155/2022/4439681. 35187164 PMC8850030

[45] Moraes ÍAP, Lima JA, Silva NM, et al. Effect of longitudinal practice in real and virtual environments on motor performance, physical activity and enjoyment in people with autism spectrum disorder: a prospective randomized crossover controlled trial. Int J Environ Res Public Health. 2022;19(22):14668. doi:10.3390/ijerph192214668. 36429386 PMC9690405

[46] Ribeiro CM, Gomes RDA, Monteiro CBM, et al. Heart rate variability during virtual reality activity in individuals after hospitalization for COVID-19: a cross-sectional control study. Electronics. 2023;12(8):1925. doi:10.3390/electronics12081925.

[47] Al Backer NB. Correlation between autism treatment evaluation checklist (ATEC) and childhood autism rating scale (CARS) in the evaluation of autism spectrum disorder. Sudan J. Paediatr. 2016;16:17. 27651549 PMC5025928

[48] Moon SJ, Hwang JS, Shin AL, et al. Accuracy of the childhood autism rating scale: a systematic review and meta‐analysis. Dev Med Child Neurol. 2019;61(9):1030–1038. doi:10.1111/dmcn.14246. 30977125

[49] van Wijk CH, Martin JH, Hans-Arendse C. Clinical utility of the Brunel mood scale in screening for post-traumatic stress risk in a military population. Mil Med. 2013;178(4):372–376. doi:10.7205/MILMED-D-12-00422. 23707819

[50] Zhang CQ, Si G, Chung PK, et al. Psychometric properties of the Brunel mood scale in Chinese adolescents and adults. J Sports Sci. 2014;32(15):1465–1476. doi:10.1080/02640414.2014.898184. 24702192

[51] Rohlfs I, Rotta TM, Luft CDB, et al. Brunel mood scale (BRUMS): an instrument for early detection of overtraining syndrome. Rev Bras Med Esporte. 2008;14(3):176–181. doi:10.1590/S1517-86922008000300003.

[52] Sties SW, Gonzáles AI, Netto AS, et al. Validation of the Brunel mood scale for cardiac rehabilitation program. Rev Bras Med Esporte. 2014;20(4):281–284. doi:10.1590/1517-86922014200401999.

[53] Jelsma D, Geuze RH, Mombarg R, et al. The impact of Wii fit intervention on dynamic balance control in children with probable developmental coordination disorder and balance problems. Hum Mov Sci. 2014;33:404–418. doi:10.1016/j.humov.2013.12.007. 24444657

[54] Yu C, Wong S, Lo F, et al. Study protocol: a randomised controlled trial study on the effect of a game-based exercise training program on promoting physical fitness and mental health in children with autism spectrum disorder. BMC Psychiatry. 2018;18(1):56. doi:10.1186/s12888-018-1635-9. 29486750 PMC5830347

[55] Zamunér AR, Moreno MA, Camargo TM, et al. Assessment of subjective perceived exertion at the anaerobic threshold with the borg CR-10 scale. J. Sports Sci.Med. 2011;10:130–136. 24149305 PMC3737915

[56] Williams N. The Borg rating of perceived exertion (RPE) scale. Occupat. Med. 2017;67(5):404–405. doi:10.1093/occmed/kqx063.

[57] Monteiro CBM, Silva TD, Abreu LC, et al. Short-term motor learning through non-immersive virtual reality task in individuals with down syndrome. BMC Neurol. 2017;17(1):71. doi:10.1186/s12883-017-0852-z. 28410583 PMC5391542

[58] Lakens D. Calculating and reporting effect sizes to facilitate cumulative science: a practical primer for t-tests and ANOVAs. Front Psychol. 2013;4:863. doi:10.3389/fpsyg.2013.00863. 24324449 PMC3840331

[59] Gidley Larson JC, Bastian AJ, Donchin O, et al. Acquisition of internal models of motor tasks in children with autism. Brain. 2008;131(Pt 11):2894–2903. doi:10.1093/brain/awn226. 18819989 PMC2577807

[60] Hayes SJ, Andrew M, Foster NC, et al. Sensorimotor learning and associated visual perception are intact but unrelated in autism spectrum disorder. Autism Res. 2018;11(2):296–304. doi:10.1002/aur.1882. 29052364

[61] Schuch JB, Müller D, Endres RG, et al. Psychomotor agitation and mood instability in patients with autism spectrum disorders: a possible effect of SLC6A4 gene? Research in Autism Spectrum Disorders. 2016;26:48–56. doi:10.1016/j.rasd.2016.03.001.

[62] Li W, Pozzo-Miller L. Dysfunction of the corticostriatal pathway in autism spectrum disorders. J Neurosci Res. 2020;98(11):2130–2147. doi:10.1002/jnr.24560. 31758607 PMC7242149

[63] Sorensen C, Zarrett N. Benefits of physical activity for adolescents with autism spectrum disorders: a comprehensive review. Rev J Autism Dev Disord. 2014;1(4):344–353. doi:10.1007/s40489-014-0027-4.

[64] Cynthia C, Duck M, McQuillan R, et al. Exploring the role of physiotherapists in the care of children with autism spectrum disorder. Phys Occup Ther Pediatr. 2019;39(6):614–628. doi:10.1080/01942638.2019.1585405. 30957621

[65] Asbury K, Fox L, Deniz E, et al. How is COVID-19 affecting the mental health of children with special educational needs and disabilities and their families. JAutism Dev Disorders. 2020;51:1772–1780. 10.1007/s10803-020-04577-2 PMC7393330 32737668

[66] Amorim R, Catarino S, Miragaia P, et al. The impact of COVID-19 on children with autism spectrum disorder. Revue Neurologique. 2020;71(8):285–291. 10.33588/rn.7108.2020381 33034366

[67] Arnell S, Jerlinder K, Lundqvist LO. Perceptions of physical activity participation among adolescents with autism spectrum disorders: a conceptual model of conditional participation. J Autism Dev Disord. 2018;48(5):1792–1802. doi:10.1007/s10803-017-3436-2. 29236210 PMC5889777

[68] Eversole M, Collins DM, Karmarkar A, et al. Leisure activity enjoyment of children with autism spectrum disorders. J Autism Dev Disord. 2016;46(1):10–20. doi:10.1007/s10803-015-2529-z. 26210514

